# Design and pilot validation of A-gear: a novel wearable dynamic arm support

**DOI:** 10.1186/s12984-015-0072-y

**Published:** 2015-09-18

**Authors:** Peter N Kooren, Alje G Dunning, Mariska M H P Janssen, Joan Lobo-Prat, Bart F J M Koopman, Micha I Paalman, Imelda J M de Groot, Just L Herder

**Affiliations:** Department of Physics and Medical Technology, VU Medical Center, Amsterdam, The Netherlands; Department of Precision & Microsystems Engineering, Delft University of Technology, Delft, The Netherlands; Department of Biomechanical Engineering, Delft University of Technology, Delft, The Netherlands; Department of Rehabilitation, Donders Center for Neuroscience, Radboud University Medical Center, Nijmegen, The Netherlands; Department of Biomechanical Engineering, University of Twente, Enschede, The Netherlands; Department of Mechanical Automation, University of Twente, Enschede, The Netherlands

## Abstract

**Background:**

Persons suffering from progressive muscular weakness, like those with Duchenne muscular dystrophy (DMD), gradually lose the ability to stand, walk and to use their arms. This hinders them from performing daily activities, social participation and being independent. Wheelchairs are used to overcome the loss of walking. However, there are currently few efficient functional substitutes to support the arms. Arm supports or robotic arms can be mounted to wheelchairs to aid in arm motion, but they are quite visible (stigmatizing), and limited in their possibilities due to their fixation to the wheelchair. The users prefer inconspicuous arm supports that are comfortable to wear and easy to control.

**Methods:**

In this paper the design, characterization, and pilot validation of a passive arm support prototype, which is worn on the body, is presented. The A-gear runs along the body from the contact surface between seat and upper legs via torso and upper arm to the forearm. Freedom of motion is accomplished by mechanical joints, which are nearly aligned with the human joints. The system compensates for the arm weight, using elastic bands for static balance, in every position of the arm. As opposed to existing devices, the proposed kinematic structure allows trunk motion and requires fewer links and less joint space without compromising balancing precision.

The functional prototype has been validated in three DMD patients, using 3D motion analysis.

**Results:**

Measurements have shown increased arm performance when the subjects were wearing the prototype. Upward and forward movements were easier to perform. The arm support is easy to put on and remove. Moreover, the device felt comfortable for the subjects. However, downward movements were more difficult, and the patients would prefer the device to be even more inconspicuous.

**Conclusion:**

The A-gear prototype is a step towards inconspicuousness and therefore well-received dynamic arm supports for people with muscular weakness.

**Electronic supplementary material:**

The online version of this article (doi:10.1186/s12984-015-0072-y) contains supplementary material, which is available to authorized users.

## Background

Duchenne Muscular Dystrophy (DMD) is the most common genetic neuromuscular disorder diagnosed in childhood, affecting approximately one in every 5000 live male births [1]. Due to the dystrophin gene being located on the X-chromosome, DMD primarily affects boys. DMD is caused by a mutation in the gene that encodes for dystrophin and results in progressive loss of muscle strength and muscle tissue [2].

People suffering from progressive muscular weakness, like those with DMD, can lose the ability to walk and stand and the ability to control the function of their arms. This hinders them from performing daily activities, participating socially and being independent. A wheelchair can overcome the loss of walking. However, for the loss of arm function there seem to be few efficient and well adopted aids. Currently used aids are powered and non-powered arm supports and robot arms mounted on the wheelchair. Overviews are given by van der Heide [3], Dunning [4] and Mahoney [5]. These overviews show for example the Armon (MicroGravity, NL), the WREX (Jaeco, US) and the Darwing (Focal, NL). The majority of the existing arm supports is mounted on the wheelchair, which limits the range of motion. Moreover, existing supports are quite visible [6] and can be experienced as stigmatizing.

In the case of boys with DMD, due to improved medical care and technical possibilities, life expectancy has increased rapidly [7, 8]. As a consequence, most of them will have no functional arm movements for more than half of their life, if unsupported.

A survey, in which 350 persons with DMD participated worldwide, stated that only a small percentage (8.5 %) of DMD patient uses an arm support. In addition, this survey describes which ADL tasks are most important for DMD patients [9]. Essential activities to perform with an arm support are eating, drinking, use of a phone and computers, personal hygiene, physical contact with others and dressing. Persons with DMD will use an arm support seated only, since they are in a wheelchair at the time they need an arm support. Wishes with respect to the arm support, apart from increased ability, are inconspicuousness, intuitive control, easy donning and comfort [6, 10]. The arm support preferably would be worn underneath clothing, e.g. sweater and pants.

Therefore, the objective of this study was to develop, and pilot test in persons with DMD, a novel wearable arm support. This paper describes a prototype design for an inconspicuous arm support for activities of daily living (ADL tasks) and presents the characterization and validation of this device.

The support is called A-gear, where the A stands for ability. The A-gear is a piece of equipment increasing the user’s ability.

## Methods

### Design method

**Fig. 1:**
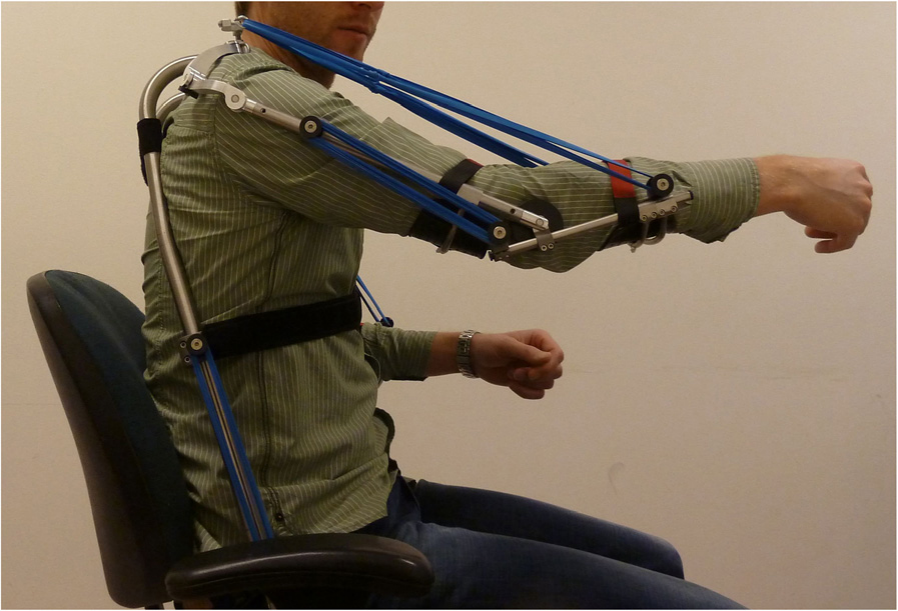
The prototype arm support worn by a healthy user

**Fig. 2:**
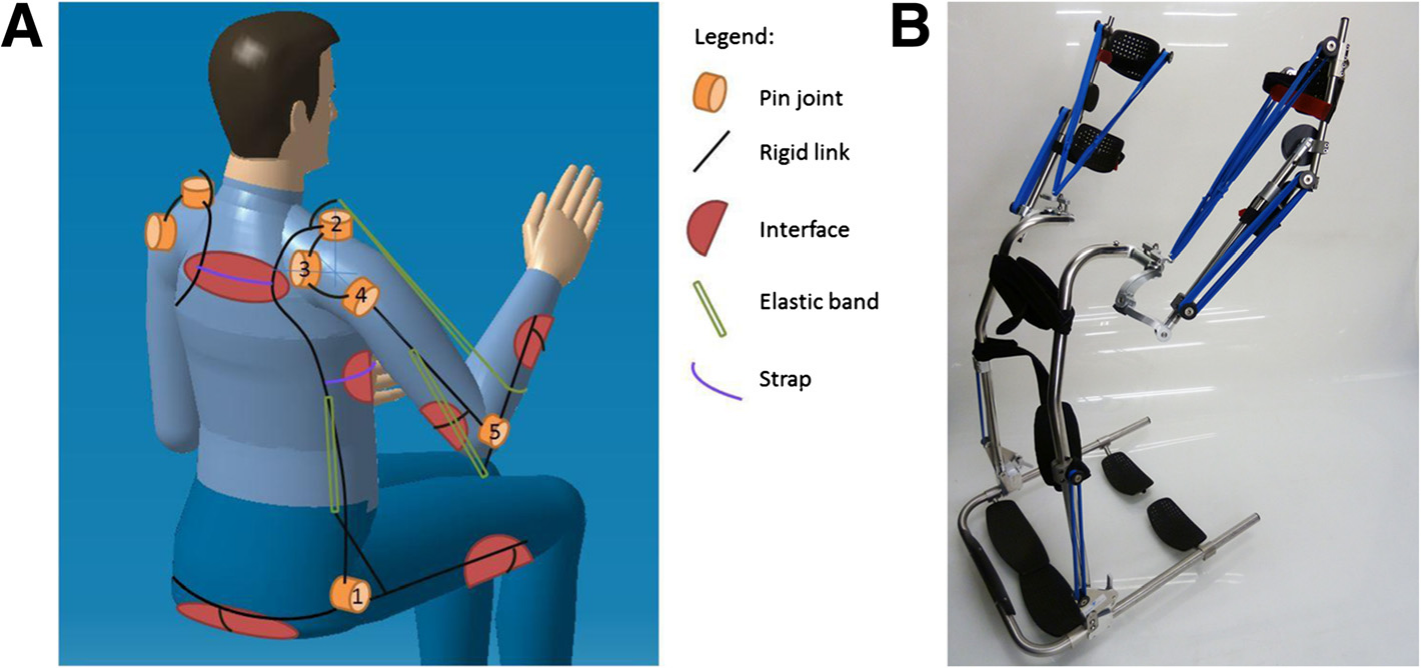
**a** A schematical representation of the kinematical architecture of the device. **b** A picture of the prototype.

To generate design concepts the main function of the device, namely to support arm motion, is split into sub functions [11]. The sub functions are: 1) generating force to compensate for the weight of the arm, 2) transferring reaction forces through the arm support and 3) transferring forces to and from the user. First, solutions were generated for these sub functions by a team of medical specialists, technical specialists and a person with DMD, resulting in a morphological overview. By systematically combining the solutions for the sub functions about 700 possible concepts could be conceived. Seven concepts were intuitively selected from the morphological overview and elaborated to realistically dimensioned sketches. These drawings helped to evaluate them within the same team of specialists and choose the optimal concept to detail and manufacture. “Optimal” meant scoring best on the combination of these criteria: low balancing error, close to the body, technical feasibility, ease of donning and comfort. These criteria resulted from the user requirements, which arose from discussion with users, their relatives and their caregivers. The optimal concept uses rubber springs for storing energy and generating the supporting force. Reaction forces are transferred through a mechanism of rigid links with pivot joints nearly aligned the human joints. This near alignment results in a support that stays close to the body and that has a range of motion (ROM) resembling human ROM, so that ADL’s can be performed. Ranges of motion of the human joints that correspond to important ADL’s were found in literature [12, 13]. The arm support interfaces with the user through perforated pads under the forearm, upper arm and underneath the upper legs. See Figs. 1 and 2.

**Fig. 3:**
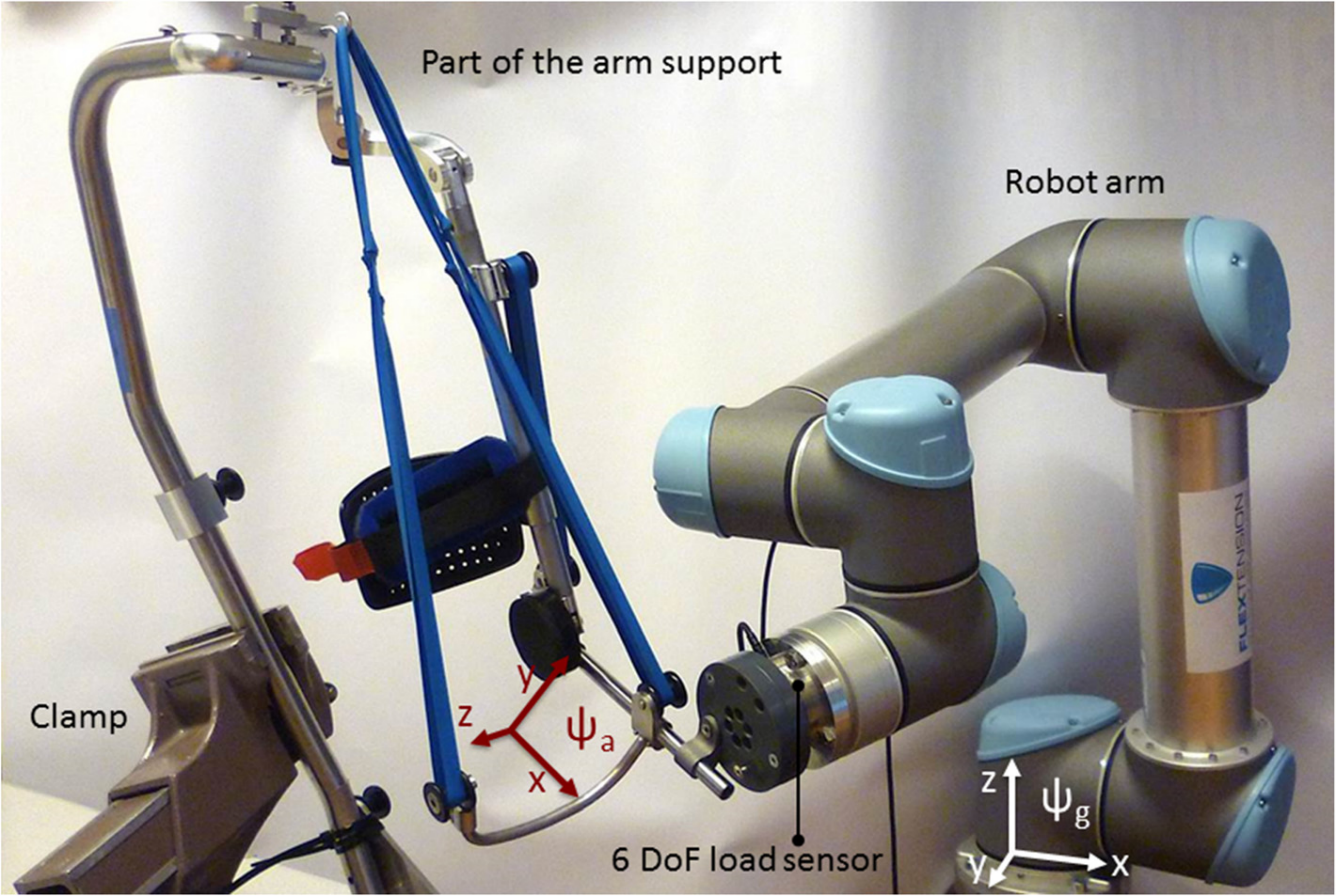
Setup for analyzing the balancing error. The balancing error of the prototype was verified by connecting it with a robot arm equipped with a six DoF load sensor

**Fig. 4:**
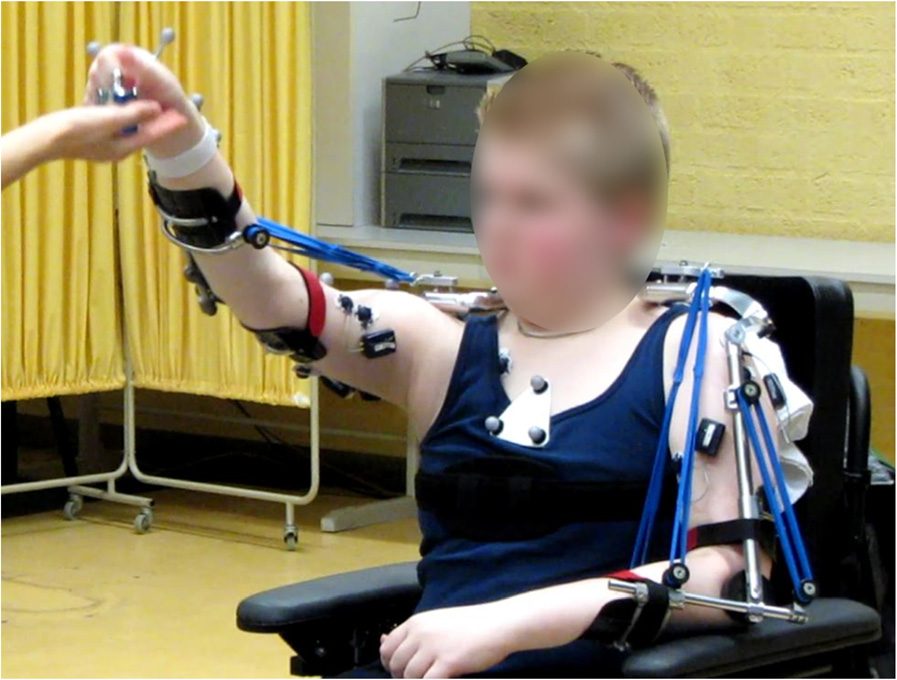
Boy with Duchenne testing the prototype, while wearing electromyography and motion capturing devices

**Fig. 5:**
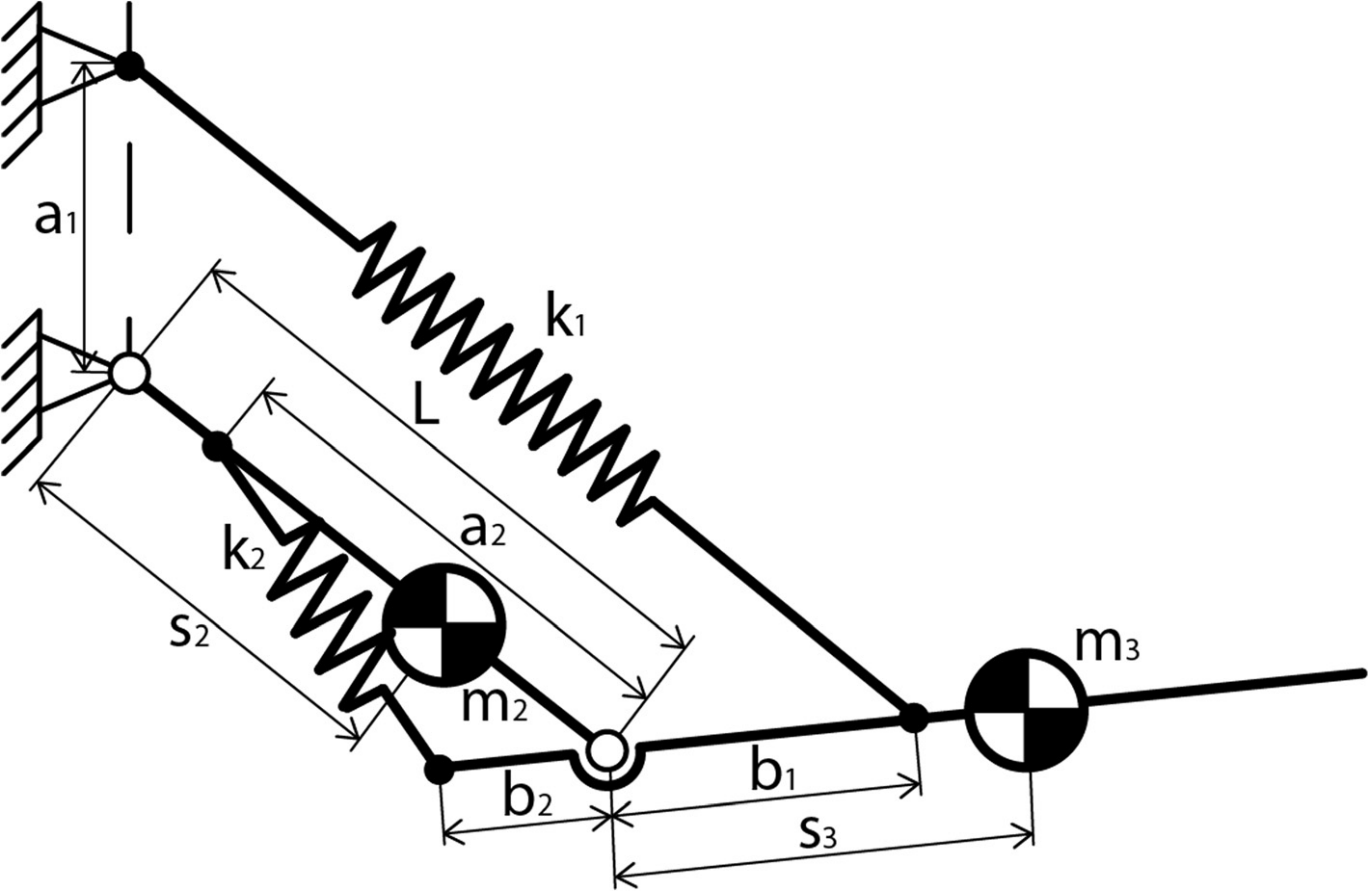
The principle of statically balancing the device. The principle and it’s parameters are described by Lin et al. [16]

**Fig. 6:**
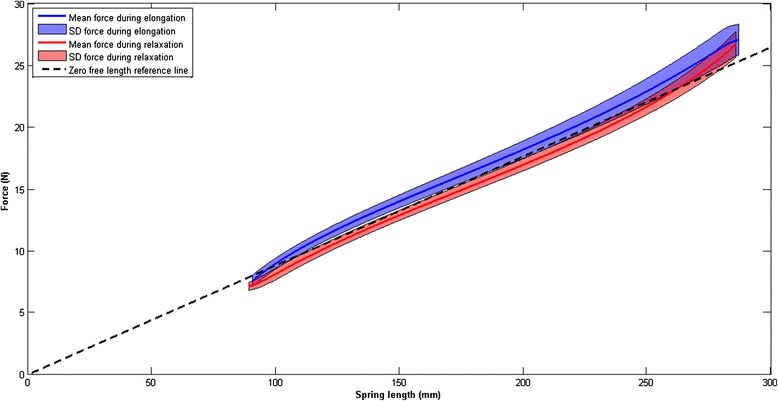
Characteristic of the rubber band with the zero-free-length spring behavior. In *blue* the mean and standard deviation of the force/displacement curve during elongation of the rubber band are shown. In *red* the same curve is shown during relaxation of the elastic band. The *black dotted line* shows the zero-free-length reference line

**Fig. 7:**
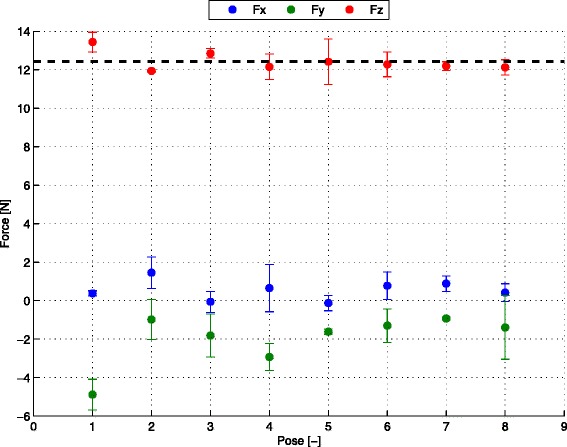
Plot of the mean measured forces exerted by the arm support with the 68 % confidence interval. The poses are shown in Fig. 10

**Fig. 8:**
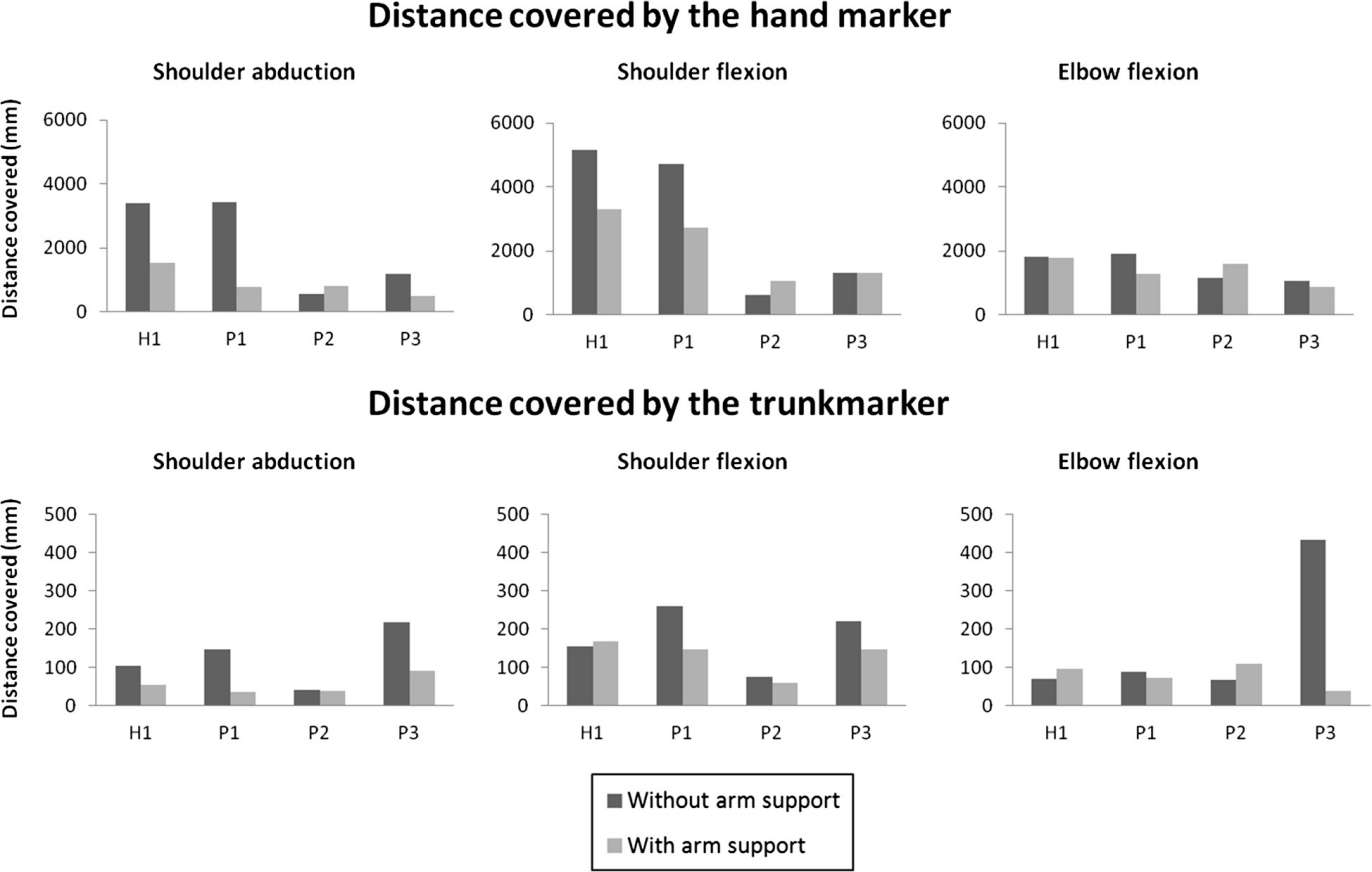
Range of motion displayed as the distance covered by the hand and trunk during single joint movements (shoulder abduction, shoulder flexion and elbow flexion), displayed for four different subjects with and without the passive arm support

**Fig. 9:**
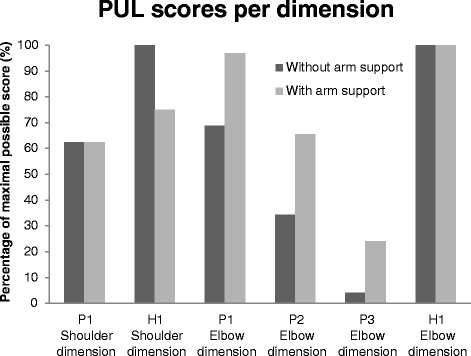
Performance of the Upper Limb scores per dimension as percentage of the maximal possible score of the dimension. P1, P2 and P3 are DMD patients, H1 is the healthy subject

### Characterization method

**Fig. 10:**
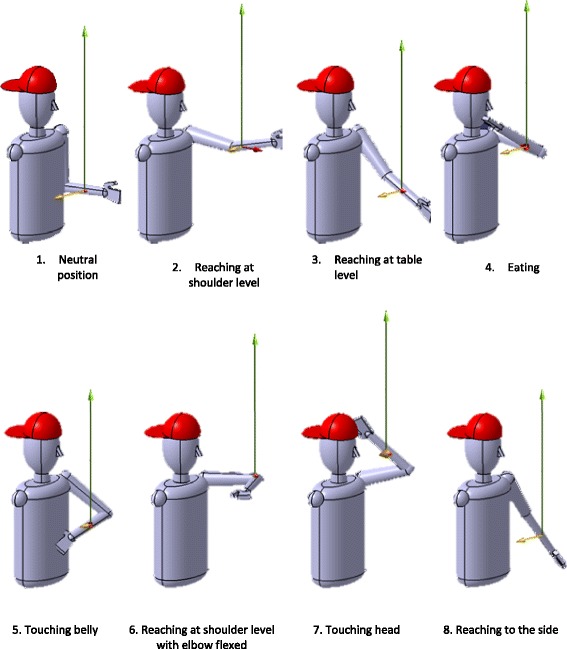
Forces exerted by the arm support displayed in context of the user. Moreover, the figure shows the poses, in which balancing error of the arm support was determined

The performance of the prototype is best characterized by the relative balancing error, E_b_.

1 $$ {E}_b=\frac{F{z}_{max}-F{z}_{min}}{F{z}_{max}+F{z}_{min}}\ast 100\% $$

where Fz_max_ and Fz_min_ represent the maximum and minimum upward forces exerted by the arm support on the virtual combined center of mass (CCOM) of the arm. To evaluate the balancing error of the arm support, a series of static measurements of the balancing forces and torques in eight functional poses have been performed. These poses, as shown in Fig. 10 in the Appendix, are in close correspondence with the most important ADL tasks as described by Janssen et al. [9]. The force/torque measurements were performed attaching the forearm link of the arm support to a six Degree of Freedom (DoF) force/torque sensor (mini45, ATI Industrial Automation, USA) that was at the same time mounted to a position controlled robotic manipulator (UR5, Universal Robots, Denmark) that served as ground (Fig. 3). By switching the manipulator to a compliant state while repositioning manually, internal stresses between arm support and manipulator were minimized. Three measurements were performed at each position. A change of the force/torque sensor coordinate system was applied to the force/torque vectors in order to express the measurements at the arm coordinate system (ψ_a_), which is located at the CCOM of the arm. Furthermore, a rotation of this coordinate system was applied in order to express the force/torque signals in the global coordinate system (ψ_g_).

### Pilot validation method

For the validation of the prototype, three DMD patients with early functional limitations in their arms (Brooke scale 2 and 3. People in scale 2 can raise their arm above the head only by flexing the elbow. People in Brooke scale 3 cannot raise their arm above the head, but can raise a filled glass to the mouth) and one healthy subject, participated in testing the prototype (see Table 1 and Fig. 4). The healthy subject was included to establish reference values for the performance with and without the prototype. Participants were included through the Radboud UMC outpatient clinic and by advertising the study on the website of a Dutch patient organization. This study was approved by the medical ethical committee Arnhem-Nijmegen, the Netherlands, and subjects and their parents gave informed consent before participating in the study.

**Table 1:** Data of subjects in the pilot validation study

Subject	Disease	Age [yrs.]	Sex	Weight [kg]	Brooke scale
1	DMD	15	M	63	2
2	DMD	14	M	70	3
3	DMD	17	M	82	3
4	Healthy	31	M	77	1

All participants performed standardized single joint movements of shoulder and elbow (shoulder flexion, shoulder abduction, shoulder horizontal adduction, shoulder internal and external rotation and elbow flexion) and ADL tasks (extracted from the shoulder and elbow dimension of the “Performance of the Upper Limb (PUL) Scale” [14], which is used to measure upper limb performance in people with DMD) with and without wearing the prototype. Examples of the tasks are stacking cans, picking up coins and tearing paper. 3D motion analysis (VICON motion analysis system (Oxford Metrics, Oxford, UK)) was performed to gain insight in the ROM of the subject, by tracking the position of the hand marker during the single joint movements. The motion data was processed with Matlab (Mathworks, Natick, USA) coded algorithms. In addition, all participants filled out a questionnaire to gain more insight in ‘functionality ‘comfort ‘aesthetics ‘safety ‘compatibility’ and ‘donning and doffing’.

## Design results

### Kinematic architecture

The arm support is supporting the forearm at the CCOM. In 3D space, the forearm of a user has six DoF’s. An assumption is made that a forearm supported by a curved interface can rotate within the skin when the user pro- or supinates the hand. Therefore, the mechanism of the arm support should provide the other five DoF’s. Intentionally, the arm support is only connected with the upper legs and forearm. In this manner, intermediate parts do not have to move synchronously with the human body and the joints do not have to be aligned perfectly. Still, near alignment is required, for the arm support to stay close to the body. An interface is placed against the upper arm, but this interface only supports the arm when the forearm is pointing upward. Without this interface the forearm would slip from the support when it is in vertical orientation with the hand upward.

Per arm, five revolute joints in series are used as kinematic chain. The first one is next to the hip. The second, third and fourth joint are pointing approximately towards the shoulder’s rotation point, and the fifth is next to the elbow (see Fig. 2). Revolute joints are simple and can be implemented with little friction. The advantage of having three joints in the shoulder region is that the arm support stays on the outer side of the arm. Therefore, the user can have direct contact with his arms on a table, and approach a table without bumping parts of the arm support against it.

Arc lengths between joint 2 and 3 and between 3 and 4 (Fig. 2) are chosen to be 56° such that the ROM of the human shoulder complex [15] is largely covered. The radius of the arcs is 70 mm. In this size, there is no interference of the arcs with the wheelchair’s back- and headrest. Revolute joint 2 is tilted 10° posteriorly and 10° medially, to comply with the human shoulder motion, and also to make space for elastic bands. During arm motion, no singularities are encountered in the shoulder joint. The ROM of the individual revolute joints is limited with end stops.

The links between the joints, which are implemented as tubes, are custom made for the intended user.

### Interfacing with user

The user is sitting on five pads (two below each upper leg, one against the user’s bottom). The pads are flexible and can be formed to the body. The pads are clicked on metal tubes, which fixate their shape. The forearm link is attached to the users arm with a pad and a Velcro band. The upper arm pad is only to prevent the forearm from slipping from its pad when pointing upward. The pad against the forearm is the dominant contact point.

Since the user is sitting in the mechanism and it is only attached to the upper and lower arm, the complete mechanism is easy to put on and take off. Moreover, since the structure runs parallel to the users arm and trunk, it has the opportunity to be worn underneath clothing.

### Static balance

The balancing concept described by Lin et al. [16] was applied to the A-gear. This concept provides a supporting force throughout the whole ROM of the human arm, combined with a slender mechanism consisting of few parts. A statically balanced system is in force equilibrium in all its possible postures. An arm that is statically balanced can therefore be moved with hardly any muscle force. In the concept of Lin, a two link mechanism with four DoF is balanced by only two springs. See Fig. 5. The first link (e.g. the upper arm) is connected with a spherical joint to a fixed point; the second link (e.g. the forearm) is connected to the first with a revolute joint. One bi-articular spring running from a point above the spherical joint (e.g. the shoulder joint) to the second link, combined with a mono-articular spring running from the first link to the second link, provides a vertical force in the combined center of gravity of both links. This force is equal in size and opposite in direction to the gravitational force of both links. The springs that are used are zero-free-length springs. The balancing force is adjusted by varying the height of the spring attachment above the shoulder, a1. The prototype design allows for this adjustment.

In order to keep the structure close to the body and to avoid a structure below the elbow, the mono-articular spring is transferred to run along the upper arm, instead of the lower arm (Fig. 5). The parameters for the spring system are calculated as described in Lin et al., and shown in Eqs. 2 to 4.

2 $$ {b}_1=\frac{m_3{s}_3L}{m_2{s}_2+{m}_3L} $$

3 $$ {k}_1=\frac{g\left({m}_2{s}_2+{m}_3L\right)}{a_1L} $$

4 $$ {k}_2=\frac{k_1{b}_1L}{a_2{b}_2} $$

The distances a1, a2 and b2 were chosen to be practical in the device. When resulting stiffness k1 and k2 could not be implemented with the available elastic bands, then the nearest feasible stiffness was chosen and a1 and b2 adjusted to satisfy the balancing criteria.

The mass of the human upper arm is divided to the shoulder and the elbow according to the position of the center of mass of the upper arm. This means that in the equations from Lin et al., to calculate the parameters of the spring system, *m*_*2*_ is only the mass of the link of the prototype along the upper arm. The combined mass *m*_*3*_ is the sum of the mass of the forearm, a part of the mass of the upper arm and the mass of the link of the prototype along the forearm (Eq. 5). According to this mass distribution the center of combined mass on the forearm is calculated using Eq. 6.

5 $$ {m}_3={m}_{FA}+{m}_{UA}\cdot \frac{s_2}{L}+{m}_{link3} $$

6 $$ {s}_3=\frac{m_{FA}\cdot {s}_{FA}+{m}_{link3}}{m_3} $$

Rubber bands are chosen above metal springs, since a certain mass or volume of rubber that is axially stretched can store more elastic energy than the same mass or volume of metal in a helical spring [17]. Consequently, the arm support will be more lightweight and slender. To find springs matching the characteristics needed to balance the arm, we have compared the characteristics of different elastic bands. The rubber bands used in the arm support (Synthetic Polyisoprene, Jaeco Orthopedic, USA), almost behave like a zero-free-length spring between 150 % and 400 % strain, as is shown in Fig. 6. To verify whether the zero-free-length reference line is indeed related to the force/displacement curve, the intraclass correlation coefficient (Two-way mixed, average measure, ICC(3,k)) was calculated. The ICC between the reference line and the average force was 0.997, meaning that the spring characteristics match the zero-free-length reference line almost perfectly. This makes these elastic bands very suitable for this application. The stiffness can be varied stepwise by changing the amount of elastic bands.

### Prototype

The manufactured prototype is shown in Figs. 1 and 2. The straight and bent tubes are made of steel, for convenient bending and welding. In future products, the tubes could be made of a composite material for weight reduction. A tube was designed, within the limits of the tube bending process, which follows the human shape as close as possible in order to be inconspicuous and fit between user and the wheelchair’s backrest.

To interface with the user, polymer pads that have padding and perforation were used for comfort purposes (Fig. 2). In existing orthotics, this type of pads has been experienced as comfortable.

## Characterization results: balancing error

The balancing error test results (Fig. 7 and Table 2 in the Appendix) show that the gravity compensation force generated by the passive arm support is nearly constant across the eight poses (Fig. 10) with a mean vertical force of 12,4 N. By considering the lowest measured vertical force (12,0 N) and the highest measured vertical force (13,4 N), the arm support presents a vertical balancing error of 6 %, using Eq. 1. Additionally, the arm support presents the maximum non-vertical norm force of 4,9 N and a maximum norm torque of 1.14 Nm.

## Results pilot validation

### Range of motion

ROM was calculated as the distance over which the hand moved during single joint movements of the shoulder and elbow. In addition, we calculated the distance over which the trunk moved during the single joint movements, to gain insight in compensatory movements of the subjects, as large trunk movements are often used to compensate for muscular weakness during daily activities. The distance, over which the hand and trunk moved during shoulder abduction, shoulder flexion and elbow flexion, are shown in Fig. 8. In addition, Additional file 1: Video 1 gives an impression of the pilot validation in one subject.

The distance, over which the hand moved during shoulder abduction and shoulder flexion, when wearing the passive arm support, decreased in the healthy subject and in two out of three patients (Fig. 8). When looking at the movement of the trunk marker we saw that this movement was reduced in all patients when wearing the passive arm support. This indicated that less compensatory movements were used when wearing the passive prototype.

Elbow ROM did not change much when wearing the passive arm support, as participants were able to flex and extend the elbow over the entire passive ROM with and without the arm support. Therefore the active elbow ROM is not limited by the arm support, but by contractures in the elbow joint, which often occur in DMD patients. One subject with minimal elbow contractures, however, experienced a bit limited elbow extension.

### Performance of the upper limb

To gain more insight in the subject’s ability to perform ADL tasks with and without the passive arm support, the participants performed tasks from the shoulder and elbow dimension of the PUL scale [14]. The healthy subject and the subject with Brooke 2 performed the items from the shoulder and elbow dimension (dimension is meant in the clinical sense not in the technical) of the PUL. The subjects with Brooke 3 only performed the elbow dimension, since they were not able to execute the items from the shoulder dimension without the prototype. Figure 9 shows the PUL scores per dimension as percentage of the maximal possible score on that dimension. The PUL scores of all patients improved for the elbow dimension, meaning that patients were able to perform more tasks and used less compensatory movements when wearing the arm support. The PUL score of the shoulder dimension of the healthy subject reduced, due to the limited shoulder ROM of the passive arm support.

### Questionnaire

The questionnaire consisted of question regarding: ‘functionality’, ‘comfort’, ‘aesthetics’, ‘safety’, ‘compatibility’ and ‘donning and doffing’.

Upwards and forward movements are experienced easier while downward movements are experienced more difficult. On average, participants felt a little limited in their ROM by the prototype. However, the subjects stated that they were all still able to perform important activities, such as drinking and reaching for objects. In addition, the participants stated that the prototype fitted well and felt comfortable. However, sometimes the shoulder parts of the prototype interfere with the shoulder of the user or the wheelchair and sometimes the arm part collided with the table or wheelchair. The lower arm interface felt comfortable to all participants. All participants stated that the arm support could not be worn underneath clothing. The opinions about the looks of the prototype differed between participants. One participant stated that he thought the visible parts of the prototype looked nice, while other participants stated that the appearance of the prototype should still be improved before they were willing to wear it in daily life. On the level of safety all patients were satisfied. The arm was steadily attached in the arm support. Furthermore, the prototype did not make unintended movements and was stable. One participant felt his skin getting pinched near the shoulder, while other participants did not have this experience. The prototype did not inhibit breathing. Donning the prototype was experienced harder than doffing the prototype, although most participants thought that the time it took to put on and off the prototype was reasonable.

Overall, all patients stated that they would like to use such an arm support in daily life, however they would also like to see some adaptations to prevent collisions with the body and surroundings and on the looks of the prototype.

## Discussion

The results of the study show a prototype design that can be worn close to the body and permits more trunk movements, a quantification of the balancing performance and outcome of tests in which people with DMD used the arm support.

In comparison with current arm supports, the A-gear is placed more naturally to the body. The device runs parallel to the arm, trunk and upper legs of the user and has mechanical joints nearly aligned with the human joints. The design makes motion more intuitive, free of singularities and the authors believe that, by optimizing the concept, the device will fit underneath clothing.

The vertical force generated by the arm support is largely constant across the measured poses. However, a balancing error of 6 % was found and the results do show non-vertical forces and torques in the system. There may be several reasons for the error and unintended forces and torques. Firstly, the springs compensate for the intrinsic mass of the device, but do not compensate for the fact that the mass is next to the human arm instead of in line with the human arm. To compensate this offset the balancing theory should be extended. Secondly, errors may arise from interaction forces between user and support on other locations than the forearm, e.g. the upper arm pad. This effect could be reduced by a forearm interface shape that prevents the forearm from slipping out and removing the upper arm pad.

One-hundred percent weight compensation is not always preferred by patients. One of the patients wanted less supporting force, which felt more comfortable to him.

In the pilot validation, all patients showed a functional improvement on the elbow dimension of the PUL scale. The improvement indicates that they were able to perform more items, or that they had to use less compensatory strategies, when wearing the passive arm support. The distance over which the trunk moved, which is a measure for the amount of compensatory movements used, also reduced in all patients, when they were using the passive arm support. The reduction of compensatory movements is very important, as compensatory movement consumes a lot of energy and therefore they restrict the endurance to perform daily activities.

The distance over which the hand marker moved reduced in three out of four subjects, when wearing the passive arm support. For the healthy subject and the patient with Brooke scale 2 (P1), this decrease in ROM was expected, because of the kinematics of the arm support, which restricted shoulder abduction beyond 90° and shoulder flexion beyond 120°. Since both the healthy subject and P1 were able to move the arm over the entire ROM without arm support, they were restricted in their shoulder movements by the passive arm support. For the patients with Brooke scale 3, we saw that the distance over which the hand moved during single joint movements increased in one patient (P2) and decreased in another patient (P3), when wearing the passive arm support. We would have expected an increase of the distance in both patients with Brooke scale 3. One possible explanation of a reduction of the distance, over which the hand was moved in P3, might lie in the amount of compensatory movements that were used by this patient, when he was not wearing the arm support. By using compensatory movements this patient was able to move the hand, but the movements were uncontrolled and not very functional, as can be seen by the lower PUL score without the arm support. Consequently a large movement of the hand marker was seen. When this patient used the passive arm support less compensatory movements were used and much more control over the movement could be executed, therefore his functional score improved.

From the items as mentioned as essential activities to perform with an arm support (eating, drinking, use of a phone and computers, personal hygiene, physical contact with others and dressing) the vast majority can be met with the prototype according to the tests. The healthy subject already reached the maximal score of the elbow dimension without wearing the passive arm support and he was still able to do this with the passive arm support.

The results of the questionnaire indicated that patients were able to perform some activities with more ease, while other activities were more difficult. Some comments were expressed regarding comfort and safety, which should be improved in a future passive arm support.

Overall the passive arm support was especially beneficial for patients with a Brooke scale of 3, those that are not able to lift their hands above their head without support. These patients showed functional improvements and indicated that arm movements became less fatiguing. All patients stated that they would like to use such an arm support in daily life; however, some aspects of the arm support would still require improvement.

The practical implementation and clinical tests taught us which aspects need further development or should be included in a wearable arm support for people with muscular weakness. Firstly, the space between the wheelchair’s arm supports is limited for the device. These arm supports are placed close to the user for sideway stability. Next to the hips the orthosis should be very slender to fit in the seat. Secondly, supporting only one arm causes a skew posture, since arm weight hanging from one shoulder is reduced. Two sided support is preferred. Thirdly, the possibility to lean forward is much appreciated. Lastly, the arm support preferably does not run between arm and trunk and does not add considerable volume underneath the forearm and elbow. Components between arm and trunk make it uncomfortable to have the arms relaxed along the trunk. Structures below the elbow clash with tabletops when moving over them.

## Conclusions

In this paper, a design of a passive dynamic arm support for persons with reduced functional abilities of their arms, more specifically, for people with Duchenne, is proposed. The architecture of the device follows human anatomy. According to the authors knowledge, the A-gear was the first device that applied the principle for static balancing, proposed by Lin [16], in orthotics. Parameters were found so that elastics bands and attachment points stay close to the user. A step forward has been made to develop an inconspicuous arm support that can be worn underneath clothing.

Three persons with DMD tested the prototype and all showed an increased PUL score with less compensatory movements, compared with not using the support. The trunk has more freedom to move as well, due to hinges next to the hips.

Subjective feedback of the users tells that the arm support is easy to put on. Arm movements forward and up become easier, movements downward and tasks on a table top are still difficult. The users would prefer the device even more inconspicuous. The users felt wearing the device was comfortable, among others because it offers free breathing.

The shown prototype is a step towards well adopted dynamics arm supports that improve participation in society, that make people with muscular weakness more independent and more able to perform important activities in daily life.

## Appendix

**Table 2:** An overview of the results of the balancing error tests

	Pose 1	Pose 2	Pose 3	Pose 4	Pose 5	Pose 6	Pose 7	Pose 8
Tx (Nm)	0,79 (0,18)	−0,59 (0,21)	−0,36 (0,69)	−0,09 (0,09)	−0,48 (0,06)	−0,75 (0,23)	−0,48 (0,03)	−0,07 (0,58)
Ty (Nm)	−0,56 (0,14)	−0,38 (0,02)	−0,73 (0,12)	0,40 (0,36)	−0,21 (0,29)	0,51 (0,29)	0,43 (0,03)	−0,88 (0,04)
Tz (Nm)	0,60 (0,04)	0,04 (0,46)	0,16 (0,36)	0,43 (0,35)	0,14 (0,04)	0,04 (0,04)	0,15 (0,01)	−0,02 (0,08)
Fx (N)	0,38 (0,14)	1,45 (0,82)	−0,06 (0,55)	0,65 (1,23)	−0,12 (0,40)	0,77 (0,70)	0,88 (0,40)	0,41 (0,46)
Fy (N)	−4,88 (0,79)	−0,98 (1,05)	−1,81 (1,11)	−2,93 (0,71)	−1,61 (0,13)	−1,30 (0,87)	−0,93 (0,09)	−1,39 (1,65)
Fz (N)	13,44 (0,51)	11,95 (0,04)	12,85 (0,25)	12,15 (0,66)	12,42 (1,19)	12,29 (0,65)	12,19 (0,23)	12,13 (0,39)
|T| (Nm)	1,14 (0,16)	0,81 (0,11)	1,05 (0,08)	0,75 (0,09)	0,59 (0,10)	0,92 (0,34)	0,66 (0,04)	1,00 (0,04)
|F| (N)	14,32 (0,46)	12,13 (0,23)	13,03 (0,05)	13,68 (1,46)	12,53 (1,19)	12,47 (0,86)	12,27 (0,25)	12,30 (0,49)

The forces and torques are exerted by the arm support (Fig. 3). The table gives the mean and 1 SD of the X, Y and Z forces and torques, expressed in the inertial frame, together with the mean and 1 SD of the corresponding norms. The poses of the measurements are shown in Fig. 10

## Additional file

Additional file 1:
**Video 1.** An impression of the second person (P2) with DMD, testing the new arm support. (MP4 81207 kb)

## Abbreviations

DoF: Degree of freedom
PUL: Performance of the upper limb
DMD: Duchenne muscular dystrophy
ADL: Activity of daily living
CCOM: Combined center of mass
SD: Standard deviation
