# Erratum to: Design and pilot validation of A-gear: a novel wearable dynamic arm support

**DOI:** 10.1186/s12984-015-0106-5

**Published:** 2015-12-04

**Authors:** Peter N. Kooren, Alje G. Dunning, Mariska M. H. P. Janssen, Joan Lobo-Prat, Bart F. J. M. Koopman, Micha I. Paalman, Imelda J. M. de Groot, Just L. Herder

**Affiliations:** Department of Physics and Medical Technology, VU Medical Center, Amsterdam, The Netherlands; Department of Precision & Microsystems Engineering, Delft University of Technology, Delft, The Netherlands; Department of Biomechanical Engineering, Delft University of Technology, Delft, The Netherlands; Department of Rehabilitation, Donders Center for Neuroscience, Radboud University Medical Center, Nijmegen, The Netherlands; Department of Biomechanical Engineering, University of Twente, Enschede, The Netherlands; Department of Mechanical Automation, University of Twente, Enschede, The Netherlands

Unfortunately, the original version of this article [1] contained an error. Equation 6 was included incorrectly: in the original equation variable s_links3_ was missing.

The correct Equation 6 can be found below:

$$ {s}_3=\frac{m_{FA}\cdot {s}_{FA}+{m}_{link3}\cdot {s}_{link3}}{m_3} $$
